# Are aphid parasitoids locally adapted to the prevalence of defensive symbionts in their hosts?

**DOI:** 10.1186/s12862-016-0811-0

**Published:** 2016-12-12

**Authors:** Christoph Vorburger, Romain Rouchet

**Affiliations:** 1 Institute of Integrative Biology, ETH Zürich, Universitätsstrasse 16, 8092 Zürich, Switzerland; 2 EAWAG, Swiss Federal Institute of Aquatic Science and Technology & Institute of Integrative Biology, Überlandstrasse 133, 8600 Dübendorf, Switzerland

**Keywords:** Aphis, Bacterial endosymbionts, Defensive symbiosis, *Hamiltonella*, Local adaptation, *Lysiphlebus*, Parasitoids, Resistance

## Abstract

**Background:**

Insect parasitoids are under strong selection to overcome their hosts’ defences. In aphids, resistance to parasitoids is largely determined by the presence or absence of protective endosymbionts such as *Hamiltonella defensa*. Hence, parasitoids may become locally adapted to the prevalence of this endosymbiont in their host populations. To address this, we collected isofemale lines of the aphid parasitoid *Lysiphlebus fabarum* from 17 sites in Switzerland and France, at which we also estimated the frequency of infection with *H. defensa* as well as other bacterial endosymbionts in five important aphid host species. The parasitoids’ ability to overcome *H. defensa*-mediated resistance was then quantified by estimating their parasitism success on a single aphid clone (*Aphis fabae fabae*) that was either uninfected or experimentally infected with one of three different isolates of *H. defensa*.

**Results:**

The five aphid species (*Aphis fabae fabae*, *A. f. cirsiiacanthoides*, *A. hederae*, *A. ruborum*, *A. urticata*) differed strongly in the relative frequencies of infection with different bacterial endosymbionts, but there was also geographic variation in symbiont prevalence. Specifically, the frequency of infection with *H. defensa* ranged from 22 to 47 % when averaged across species. Parasitoids from sites with a high prevalence of *H. defensa* tended to be more infective on aphids possessing *H. defensa*, but this relationship was not significant, thus providing no conclusive evidence that *L. fabarum* is locally adapted to the occurrence of *H. defensa*. On the other hand, we observed a strong interaction between parasitoid line and *H. defensa* isolate on parasitism success, indicative of a high specificity of symbiont-conferred resistance.

**Conclusions:**

This study is the first, to our knowledge, to test for local adaptation of parasitoids to the frequency of defensive symbionts in their hosts. While it yielded useful information on the occurrence of facultative endosymbionts in several important host species of *L. fabarum*, it provided no clear evidence that parasitoids from sites with a high prevalence of *H. defensa* are better able to overcome *H. defensa*-conferred resistance. The strong genetic specificity in their interaction suggests that it may be more important for parasitoids to adapt to the particular strains of *H. defensa* in their host populations than to the general prevalence of this symbiont, and it highlights the important role symbionts can play in mediating host-parasitoid coevolution.

**Electronic supplementary material:**

The online version of this article (doi:10.1186/s12862-016-0811-0) contains supplementary material, which is available to authorized users.

## Background

For many insects, parasitoids are important natural enemies that cause substantial mortality, thus exerting intense selection for host resistance [1]. The hosts’ defences, in turn, impose strong selection on parasitoid infectivity. This sets the scene for antagonistic coevolution by reciprocal adaptation [2], which requires genetic variation for traits involved in the outcome of host-parasite interactions and may lead to local adaptation. Variation for parasitoid infectivity and/or host resistance was described for numerous insect host-parasitoid interactions [3–8], but studies of parasitoid local adaptation are relatively few [9–13]. For example, Dupas *et al*. [14] reported strong evidence for local adaptation of the parasitoid *Cotesia sesamiae* to immune resistance in the local host community, whereas van Nouhuys et al. [15] found that the parasitoid wasp *Cotesia melitaearum* did not perform better on local populations of its host butterfly *Melitaea cinxia*.

Parasitoid wasps are also important natural enemies of aphids and employed frequently in biological control of pest aphids [16]. Although aphids exhibit host-encoded variation for resistance to parasitoids as well [8, 17, 18], most of this variation is explained by their facultative association with bacterial endosymbionts. One of these, the gammaproteobacterium *Hamiltonella defensa* [19], has been shown to strongly increase the resistance to parasitoid wasps in the pea aphid *Acyrthosiphon pisum* [20, 21], in the black bean aphid *Aphis fabae* [22, 23], in the cowpea aphid *Aphis craccivora* [24], and presumably other aphids as well (but see [25]). The protection against parasitoids is correlated with the presence of toxin-encoding bacteriophages called APSE in the *H. defensa* genome [26–28], suggesting that these phage-derived toxins may prevent the development of the parasitoids’ eggs or early larvae. Interestingly, different strains of *H. defensa* are associated with different APSE variants and provide different levels of protection against parasitoids [26]. Other facultative endosymbionts of aphids include *Regiella insecticola*, *Serratia symbiotica* and a bacterium referred to as X-type or PAXS, as well as bacteria from the genera *Rickettsia*, *Rickettsiella*, *Spiroplasma* and *Arsenophonus* [19, 29–35]. Aphids can thus harbour entire communities of facultative endosymbionts that contribute to heritable variation in ecologically relevant traits of their hosts [36]. Their important role in aphid ecology is increasingly acknowledged [37], but there is still much to be learnt about their precise functions and about their occurrence in natural populations of aphids. Although particular strains that are protective against parasitoids have been detected in other endosymbionts as well, e.g. in *R. insecticola* [38] or *S. symbiotica* [21], none of them is consistently associated with resistance to parasitoids as is the case for *H. defensa*.

The black bean aphid, *Aphis fabae*, and its most important parasitoid, *Lysiphlebus fabarum*, have become a useful model to investigate the role of *H. defensa* in mediating aphid host-parasitoid interactions [22, 39]. Different lines of *L. fabarum* vary in their ability to parasitize aphids harbouring *H. defensa* [23, 40], indicating that parasitoid populations possess the genetic variation to overcome symbiont-conferred resistance. Using an experimental evolution approach, it has indeed been shown that *L. fabarum* is able to adapt to the presence of *H. defensa* in its host *A. fabae* [41]. Taken together, these results suggest that parasitoid wasps might have the potential to locally adapt to the prevalence of *H. defensa* in their hosts. In this study we addressed this possibility by estimating the prevalence of *H. defensa* (as well as other bacterial endosymbionts) in the main hosts of *L. fabarum* at 17 locations, and by collecting the parasitoids from the same locations to estimate their ability to overcome the protection conferred by three different strains of *H. defensa*.

## Methods

### Aphid and parasitoid collection

From May to July 2009, we simultaneously collected aphids belonging to five taxa and their parasitoids from the *L. fabarum* group at fourteen sites in Switzerland and three sites in France (Additional file 1: Table S1), with a minimum distance of 35 km between sites. A map of all sampling sites is provided in Rouchet [42]. The five aphid taxa comprised *Aphis fabae fabae*, *A. fabae cirsiiacanthoides*, *A. hederae*, *A. ruborum* and *A. urticata*. They are among the most important hosts of *L. fabarum* [43], and they can typically be found in close proximity in ruderal habitats. *Aphis hederae* was collected from the common ivy *Hedera helix*, *A. ruborum* from the blackberry shrub *Rubus fruticosus* and *A. urticata* from the stinging nettle *Urtica dioica. Aphis fabae fabae* was collected from the white goosefoot *Chenopodium album*, and *A. fabae cirsiiacanthoides* from thistles of the genus *Cirsium*, mostly *Ci. arvense* with a few individuals from *Ci. vulgare* (29 out of 290). Taxonomically, *A. f. fabae* and *A. f. cirsiiacanthoides* are typically treated as subspecies of *A. fabae* [44]. They are near-indistinguishable morphologically but they differ in the host plants used over summer [45] and show strong nuclear genetic differentiation (Vorburger C, Herzog J, Rouchet R: Aphid specialization on different summer hosts is associated with strong genetic differentiation and unequal symbiont communities despite a common mating habitat, submitted). For simplicity we will also refer to them as species in this paper.

Potential host plants were checked for aphid colonies and one individual was collected from each infested plant, with a minimum distance of 5 m between two colonies of the same species to avoid collecting clonal descendants of the same individual. The goal was to collect at least 20 aphids from each taxon per site, although this was not quite achieved for all sites (Additional file 1: Table S1). Aphids were collected into individual tubes and stored at −80 °C until DNA extraction. Parasitoids were sampled from the exact same locations and at the same time as the aphids. We collected visibly parasitized colonies of the five focal aphid species into ventilated plastic tubes (5 × 10 cm), although not all sites yielded parasitized aphids of all five species. Tubes were brought back to the laboratory and checked every second day for emerged adult parasitoid wasps. We used acetyl acetate vapour to lightly anesthetize the wasps before determining the species and sex. Two *L. fabarum* females per aphid colony were individually transferred to a caged colony of a *H. defensa*-free clone of *A. f. fabae* growing on broad bean, *Vicia faba*, to establish isofemale lines of parasitoids for later testing. Additional parasitoids emerging from the same aphid colonies were conserved in 96 % ethanol to determine their sex ratio and thus infer reproductive mode. In *L. fabarum*, sexual (arrhenotokous) as well as asexual (thelytokous) lines occur [46–48]. All-female broods are indicative of thelytoky, which is the more common mode of reproduction in *L. fabarum*. Unfortunately, a substantial fraction of parasitoids failed to establish in the laboratory. Of 223 samples from which *L. fabarum* emerged, we managed to establish at least one line in 103 cases (Additional file 2: Table S2), and the majority of them were thelytokous (92 of 103).

### Aphid DNA extraction and symbiont detection

We used a multiplex PCR assay as described  (Vorburger C, Herzog J, Rouchet R: Aphid specialization on different summer hosts is associated with strong genetic differentiation and unequal symbiont communities despite a common mating habitat, submitted) to test our aphid samples simultaneously for the presence of five facultative endosymbionts, including *H. defensa*, *R. insecticola*, *S. symbiotica*, X-type and *Rickettsia* sp. Briefly, aphid DNA was prepared using a Chelex protocol (Bio-Rad, Hercules, CA, USA), followed by amplification of part of the bacterial 16S rRNA gene with the universal bacterial forward primer 16SA1 [49] and symbiont-specific backward primers labelled with different fluorescent dyes. These produced unique combinations of fragment size and fluorescence color for each endosymbiont species that could be visualized and scored on an ABI 3730 automated sequencer. The multiplex PCR reaction also included a specific reverse primer for the obligate aphid endosymbiont *B. aphidicola*, which is possessed by all aphids and thus served as an internal positive control for the presence of amplifiable endosymbiont DNA in the DNA preparations. Samples for which *B. aphidicola* could not be detected were excluded from all analyses.

### Microsatellite genotyping of parasitoid lines

Because a large proportion of *L. fabarum* reproduce asexually, it is possible that we collected multiple parasitoids belonging to the same asexual lines either from the same or even from different geographic locations. We determined to what extent this was the case by genotyping each line with 12 microsatellites [50, 51]. When a parasitoid line was tested, one of the female wasps was collected into a 1.5 ml tube and stored at −80 °C until use. DNA extractions and microsatellite genotyping followed protocols published in Sandrock *et al*. (2007). Fragment sizes were determined on an ABI 3730 sequencer and allele scoring was done with the software GeneMapper® version 3.7.

### Parasitoid infectivity tests

To meaningfully estimate the parasitoids’ ability to overcome symbiont-conferred resistance, it is important to distinguish the protection by *H. defensa* from any underlying genetic variation in the aphid host. This was achieved by using genetically identical aphids, that is a single clone of *A. fabae fabae* (lab ID A06-407), of which we had one subline without any facultative endosymbionts and three sublines that were infected with different isolates of *H. defensa*. The infected sublines were labelled A06-407^H323^, A06-407^H402^ and A06-407^H76^. Details on the creation and the parasitoid resistance of these artificially infected lines can be found in Rouchet & Vorburger [41]. The presence of *H. defensa* in these sublines was confirmed by diagnostic PCR before the beginning of the experiments described below as well as at the end of the experiments. To measure their ability to overcome symbiont-mediated protection, we determined the infectivity of all parasitoid lines we managed to establish from field samples on the symbiont-free and the *H. defensa*-infected sublines of clone A06-407. As in Henter & Via [5], the assay consisted of exposing a group of aphid nymphs to wasps for a fixed period of time and in later counting the number of individuals that were successfully parasitized, which is easily recognized when aphids are killed by the parasitoids and turn into ‘mummies’ containing a cocoon with the metamorphosing wasps. The proportion of mummified aphids among all aphids originally exposed to the wasps was used as an estimate of parasitoid infectivity. The parasitoid lines were tested at the second, third, or fourth generation after establishment in the laboratory. If we obtained a laboratory line from both females originally taken from the same field sample, i.e. the same aphid colony, only one of the two lines was tested because it is likely that two females emerging from the same aphid colony are sisters. Each of the 103 wasp lines was tested on all four aphid sublines in five replicate assays, unless a line yielded fewer than 20 female wasps at the time of testing (mean number of replicates per parasitoid line/aphid subline combination = 4.24, minimum = 2). Each replicate was started by placing three mature aphid females on a *V. faba* seedling for 24 h to reproduce. Plants were grown in 0.07-L-plastic pots covered with a cylindrical cage. Two days after adult removal, the aphid nymphs (48 to 72 h old) were counted on each plant (mean colony size 21.5 ± 6.8 SD) and one female wasp was then introduced into the aphid colony and allowed to oviposit for 12 h. Some aphids may thus have been attacked more than once, even though aphid parasitoids generally have an (imperfect) ability to avoid self-superparasitism [52, 53]. Ten to eleven days after wasp exposure, all successfully parasitized aphids had turned into mummies and were counted.

### Statistical analysis

The effects of aphid host species and sampling site on infection with each of the facultative endosymbionts we screened for were analyzed using a generalized linear model (GLM) with a logit link function and binomial errors in R version 3.1.3 [54]. To avoid problems with non-convergence, these analyses were restricted to the 14 sites for which sufficient samples of all five aphid species could be obtained (see Additional file 1: Table S1). For each aphid species we used Fisher’s exact tests to assess whether co-infections with specific combinations of symbionts occurred more or less frequently than expected by chance based on their individual frequencies. These tests were restricted to cases where the expected frequencies of double infections were > 5.

For the tests of parasitoid infectivity on the four aphid sublines with and without different strains of *H. defensa*, the proportion of aphids that were mummified (i.e. successfully parasitized) was taken as the response variable. Severe overdispersion prevented us from analyzing these success-failure data with a generalized linear models and binomial errors. Hence, the proportions of aphids mummified were arcsine square-root transformed and analyzed with linear mixed models (LMM), using the lmer function from the lme4 package in R [55]. We tested for the effects of *H. defensa* strain (fixed), the host species parasitoids were collected from (fixed), collection site (random), and parasitoid line (random, nested within host species × collection site) as well as all possible interactions. *P*-values for the fixed effects were calculated using *F*-tests with Satterthwaite’s approximation and *P*-values for the random effects were calculated based on Chi-squared test with the lmerTest package [56].

## Results

### Secondary endosymbionts in the five *Aphis* species

**Fig. 1:**
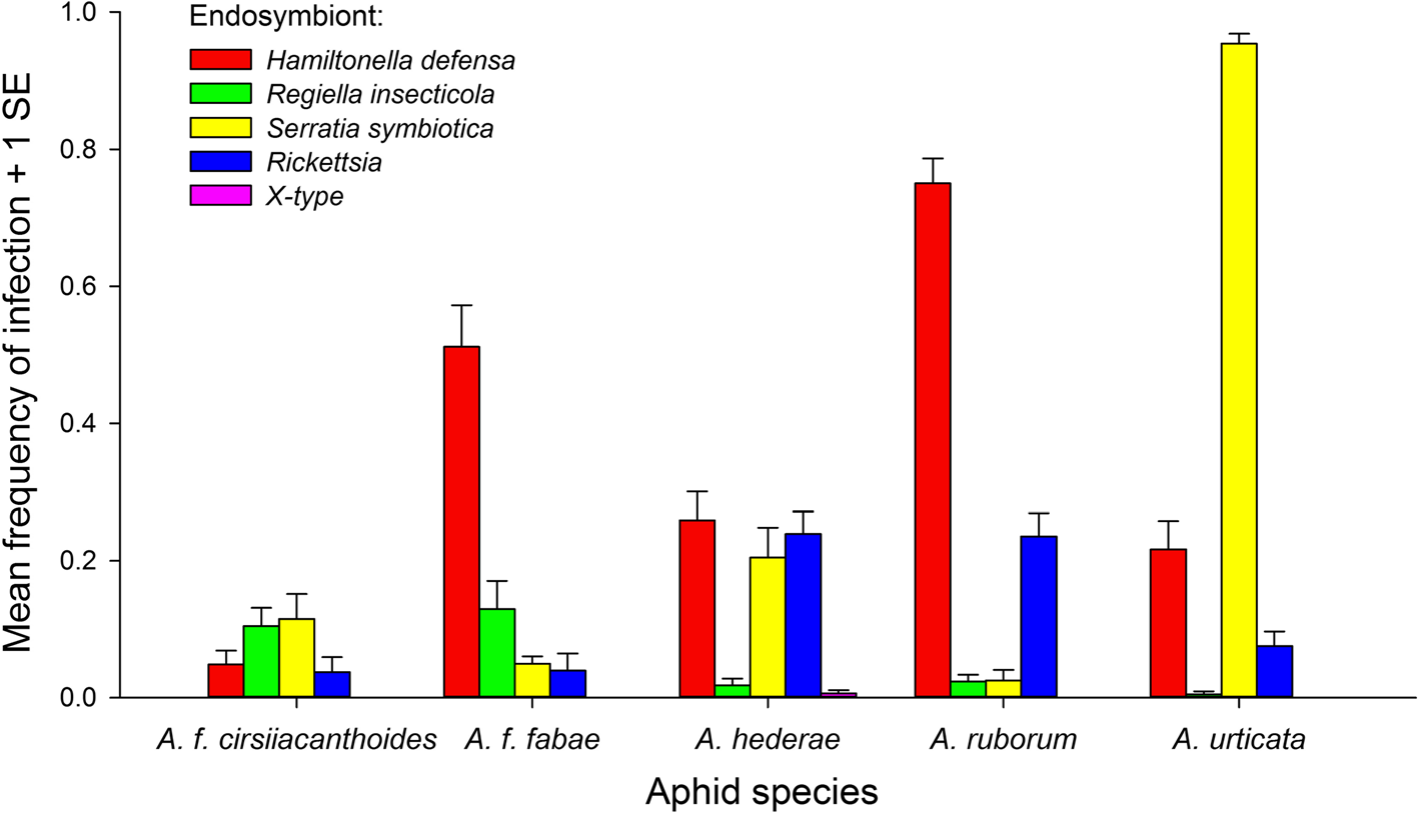
Average frequencies of infection with five secondary endosymbionts (averaged across sites) in the different aphid species analyzed in this study

Of the five secondary endosymbionts we tested for, the most common were *H. defensa*, *S. symbiotica*, *Rickettsia* and *R. insecticola* with infection rates over all individuals tested of 36.0 %, 30.5 %, 8.2 % and 6.3 %, respectively. These symbionts were present in all five species (Fig. 1). The X-type symbiont was only found in two individuals of *A. hederae* and was not analyzed further, but we did confirm the identity of this rare symbiont by sequencing part of the 16S ribosomal RNA gene (GenBank accession nrs. KX531113 and KX531114). The frequencies of infection with the four more common symbionts differed strongly among species (Fig. 1, Table 1). Most striking were the very high prevalence of *S. symbiotica* in *A. urticata* (95.4 %), as well as the high frequencies of infection with *H. defensa* in *A. ruborum* (75.4 %) and *A. f. fabae* (51.3 %) (Fig. 1). The average frequencies of infection with the four common symbionts also differed significantly among sampling sites, and a significant aphid species × site interaction for *H. defensa*, *S. symbiotica* and *Rickettsia* suggested that their frequencies in different geographic regions varied independently in the different aphid species (Table 1).

**Table 1:** Generalized linear model results for the presence/absence of the four more common facultative bacterial endosymbionts tested for in field-collected aphids

Symbiont species	Source	d.f.	Deviance	*P*
*H. defensa*	Aphid species	4	367.0	<0.001
	Site	13	54.4	<0.001
	Aphid species × site	52	198.5	<0.001
	Residual	1303	1182.0	
*R. insecticola*	Aphid species	4	60.5	<0.001
	Site	13	55.9	<0.001
	Aphid species × site	52	65.0	0.107
	Residual	1304	400.7	
*S. symbiotica*	Aphid species	4	740.8	<0.001
	Site	13	27.0	0.012
	Aphid species × site	52	97.1	<0.001
	Residual	1304	799.0	
*Rickettsia*	Aphid species	4	107.9	<0.001
	Site	13	55.8	<0.001
	Aphid species × site	52	90.7	<0.001
	Residual	1303	536.2	

We found numerous double infections with two different facultative endosymbionts of various combinations in all aphid species, as well as a few triple infections with *H. defensa*, *Rickettsia* and *S. symbiotica* in both *A. hederae* (2 individuals) and *A. urticata* (3 individuals), and one individual of *A. hederae* even harboured all four common secondary endosymbionts. Most double infections occurred approximately at the frequencies one would expect by chance based on individual frequencies of each symbiont, but three pairwise combinations occurred less often than expected by chance: *H. defensa* with *R. insecticola* in *A. f. fabae* and *A. ruborum*, as well as *H. defensa* with *S. symbiotica* in *A. ruborum* (Table 2). These results should be interpreted cautiously, however, because the expectations were calculated under the assumption of spatially homogeneous infection frequencies, which was generally not the case (Table 1).

**Table 2:** Comparisons of observed and expected frequencies of co-infections with two different secondary symbionts for all cases with an expected frequency of co-infection > 5 in an aphid species

Aphid species	Symbiont 1	Symbiont 2	observed	expected	*P*-value
*A. fabae fabae*	*H. defensa*	*Rickettsia*	6	8.2	0.312
*A. fabae fabae*	*H. defensa*	*R. insecticola*	1	26.3	<**0.001**
*A. fabae fabae*	*H. defensa*	*S. symbiotica*	9	10.2	0.504
*A. hederae*	*H. defensa*	*Rickettsia*	14	19.6	0.098
*A. hederae*	*H. defensa*	*S. symbiotica*	11	16.2	0.107
*A. hederae*	*Rickettsia*	*S. symbiotica*	11	14.6	0.246
*A. ruborum*	*H. defensa*	*R. insecticola*	0	5.3	<**0.001**
*A. ruborum*	*H. defensa*	*S. symbiotica*	30	45.1	<**0.001**
*A. urticata*	*H. defensa*	*Rickettsia*	3	6.0	0.225
*A. urticata*	*H. defensa*	*S. symbiotica*	69	69.7	0.751
*A. urticata*	*Rickettsia*	*S. symbiotica*	26	25.8	1.000

Significant deviations from expectations are indicated by bold *P*-values (Fisher’s exact tests), and these remain significant at a Bonferroni-corrected significance level α of 0.0045

### Infectivity test of the parasitoids

**Fig. 2:**
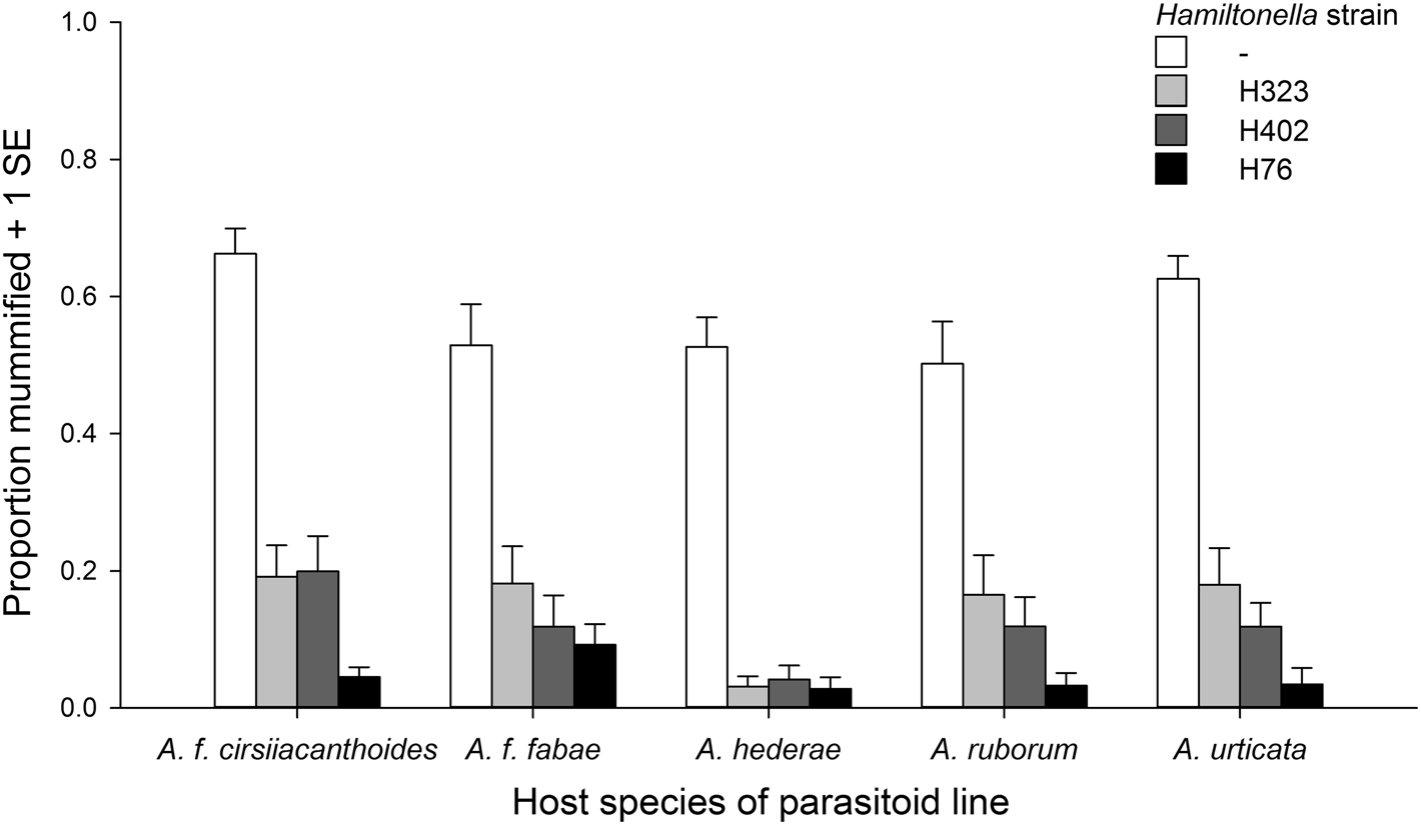
Mean infectivity of parasitoids (*Lysiphlebus fabarum*) collected from five host species on sublines of a single clone of *Aphis fabae* that were either uninfected (-) or experimentally infected with three different strains of the defensive endosymbiont *Hamiltonella defensa* (H323, H402; H76)

**Fig. 3:**
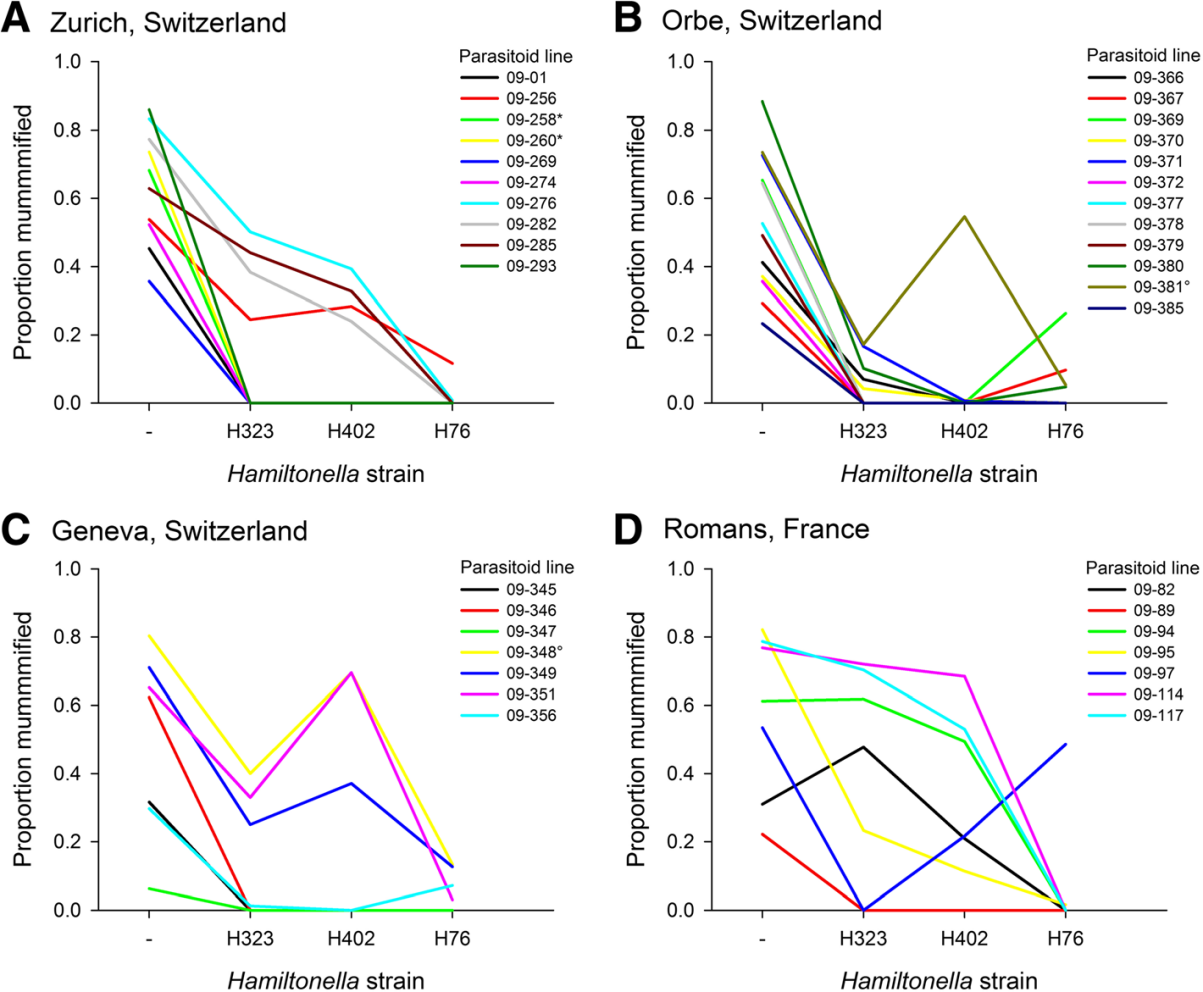
Interaction plots depicting the infectivities of multiple field-collected isofemale lines of the parasitoid *Lysiphlebus fabarum *from each of four collection sites, when tested on genetically identical aphids that were either uninfected (-) or experimentally infected with three different strains of the defensive endosymbiont *Hamiltonella defensa *(H323, H402; H76). Parasitoid lines belonging to the same asexual lineage (identical microsatellite genotypes) are marked with the same superscript symbols

There was significant variation among the four aphid sublines with and without different *H. defensa* strains in the proportion of individuals mummified by parasitoids (Table 3). As expected, the three *H. defensa*-protected sublines were much more resistant than the subline without *H. defensa* (Fig. 2). The parasitoids’ site of origin did not significantly affect the proportion of aphids mummified, and the site × *H. defensa* strain interaction was not significant, either, albeit marginally so (Table 3). The aphid host from which parasitoids were collected did not have a significant effect on mummification, but there was a significant *H. defensa* strain × host species interaction (Table 3), largely reflecting that parasitoids collected from *A. hederae* were particularly ineffective at parasitizing the *H. defensa*-protected sublines compared to parasitoids collected from other hosts (Fig. 2). Among the strongest effects in the analysis were the variation among parasitoid lines and particularly the *H. defensa* strain × parasitoid line interaction (Table 3). The 103 lines of *L. fabarum* tested varied in their ability to overcome *H. defensa*-conferred resistance, and they differed strongly in their relative infectivities on aphids protected by different strains of *H. defensa*, which is clearly visible in the interaction plots shown in Fig. 3. Interestingly, *H. defensa* strain H76 appeared to represent a very different challenge to parasitoids than strains H323 and H402 (Figs. 2 and 3). This was supported by the fact that we found a significant positive correlation between the parasitoid lines’ mean infectivities on aphid subline A06-407^H323^ and A06-407^H402^ (*r* = 0.838, *P* < 0.001), whereas parasitoid infectivity on subline A06-407^H76^ was not significantly correlated with infectivity on either A06-407^H323^ (*r* = 0.062, *P* = 0.532) or A06-407^H402^ (*r* = 0.139, *P* = 0.161). Thus, different strains of *H. defensa* provide different levels of protection to aphids depending on which parasitoid line they are attacked by.

**Table 3:** Results of the linear mixed model on the proportion of aphids mummified by parasitoids

A: Fixed effects:	ndf, ddf	*F*	*P*
* H. defensa* strain	3, 44.3	162.45	<0.001
Host species	4, 27.3	2.37	0.077
* H. defensa* str. × Host species	12, 292.6	2.31	0.008
B: Random effects:	df	χ^2^	*P*
Site	1	0.00	1.000
Site × *H. defensa* str.	1	3.72	0.054
Site × Host species	1	0.58	0.446
Site × Host species × *H. defensa* str.	1	0.00	1.000
Parasitoid line (Site × Host species)	1	37.40	<0.001
* H. defensa* str. × Parasitoid line (Site × Host sp.)	1	172.00	<0.001

Proportions were arcsine square-root transformed before analysis. *P*-values of random effects are based on likelihood ratio tests and *P*-values of fixed effects on *F* tests with Satterthwaite’s approximation [56]

In seven cases, we had collected and tested parasitoid lines with identical multilocus microsatellite genotypes either twice or three times (see Additional file 3: Table S3). In these cases, lines with identical genotypes produced very similar patterns of infectivity, for example lines 09–258 and 09–260 collected in Zurich, Switzerland, or lines 09–348 and 09–381 collected in Geneva and Orbe, Switzerland, which are depicted in Fig. 3. Hence, the ability of parasitoids to successfully infect aphids protected by a particular strain of *H. defensa* appears to be a genetically determined trait in *L. fabarum*, and this leads to a high degree of specificity in the interaction between *H. defensa* and *L. fabarum* in this system.

**Fig. 4:**
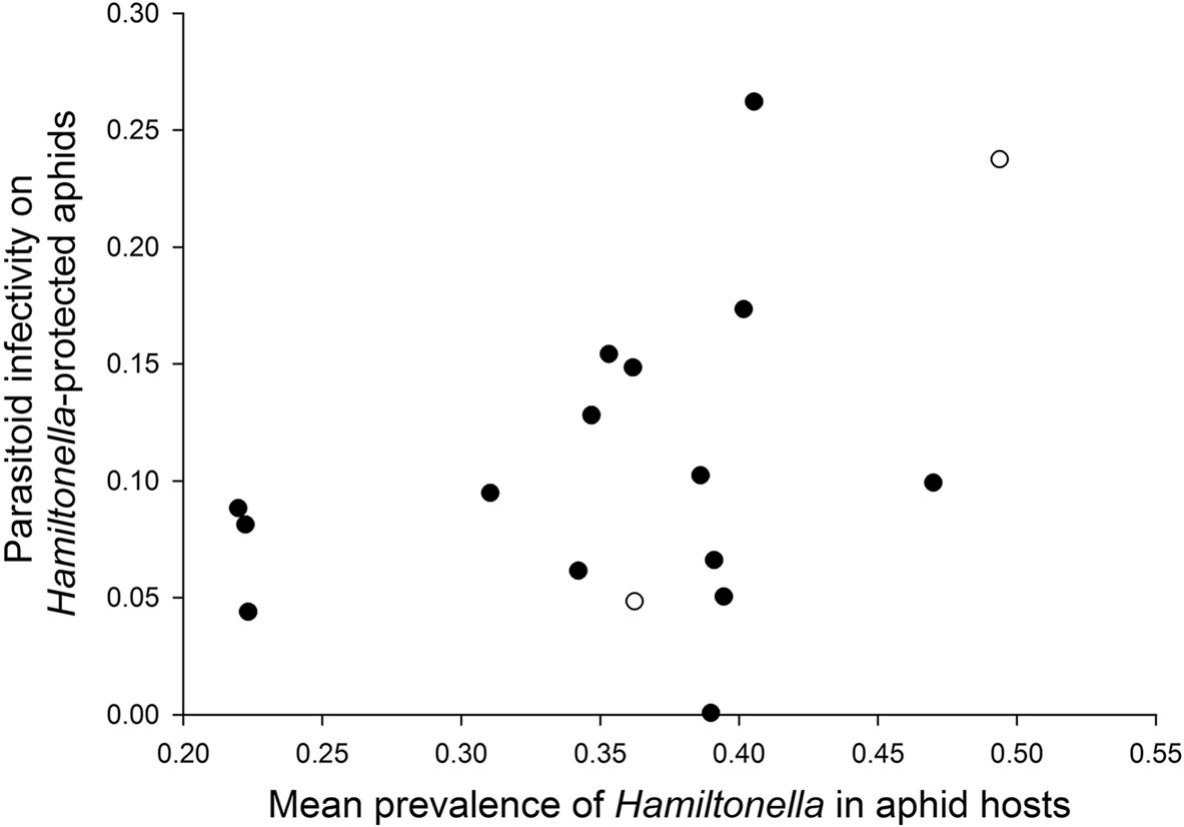
Relationship between the average infectivity of *Lysiphlebus fabarum* parasitoids from each site on *Hamiltonella defensa*-protected aphids and the mean frequency of infection with *H. defensa* across five aphid host species collected at the same sites. The two open symbols represent sites for which only a single parasitoid line could be tested. For other sample sizes please refer to Additional file 2: Table S2

To test for local adaptation of parasitoids, we related the average infectivity on *H. defensa*-protected aphids of all parasitoid lines from a given site to the average prevalence of *H. defensa* across the five aphid species collected from the same sites. In four cases, we had missing or insufficient data for the frequency of *H. defensa* in one of the aphid species. Because the frequencies of infection with *H. defensa* differed strongly among aphid species (Fig. 1), this could introduce substantial biases. Hence we replaced these missing values by the average frequency of *H. defensa* in that species before calculating averages (see Additional file 4: Table S4). Although we detected a positive relationship between the local prevalence of *H. defensa* and parasitoid infectivity on *H. defensa*-protected aphids, this correlation was not statistically significant (*r* = 0.409, *P* = 0.103) (Fig. 4), and it remained non-significant when two sites were excluded for which only one parasitoid line could be tested (*r* = 0.264, *P* = 0.341). Hence, there is no conclusive evidence that parasitoid ability to overcome *H. defensa*-mediated defences has evolved to match the local prevalence of this symbiont in their host populations.

## Discussion

We screened large field samples of five aphid species, all important hosts of the aphid parasitoid *L. fabarum*, for the presence of five facultative bacterial endosymbionts, and found that the frequencies of infection with these symbionts differed markedly among species. The symbiont communities thus appear to evolve independently in the different aphid species, even though they tend to comprise the same members. This matches observations from pea aphids, in which multiple reproductively isolated host races differ strongly in the relative frequencies of infection with different facultative endosymbionts [57, 58]. There is increasing evidence that such differences are adaptive [59–61], and it will be interesting to determine the main selective forces shaping these symbiont communities. By using diagnostic PCR, we could only detect symbionts we screened for, hence our study cannot provide a comprehensive picture of the complete symbiont community in these aphid species. For example, we did not test for the presence of *Arsenophonus*, a bacterial endosymbiont recently found to be quite prevalent in the genus *Aphis* [29].

In addition to among-species variation, the prevalence of the different bacterial endosymbionts also showed significant geographic variation. Of specific interest here: the frequency of infection with the defensive endosymbiont *H. defensa* varied from 22 to 47 % when averaged across species. It is further known that different genotypes of *L. fabarum* vary strongly in their ability to parasitize *H. defensa*-protected aphids [23, 40, 62], which was also evident in the > 100 isofemale lines we tested in the present study. Taken together, these findings indicate the potential for parasitoid local adaptation, and we hypothesized that *L. fabarum* from sites where *H. defensa* is more common in its main hosts might show an improved ability to parasitize aphids protected by this defensive symbiont. However, there was no conclusive evidence for this hypothesis from our experiment. Although there was a tendency towards higher infectivities on protected aphids when parasitoids came from sites where *H. defensa* was more prevalent, the correlation was not significant.

A possible explanation for this lack of evidence for local adaptation are the strong *H. defensa* genotype-by-parasitoid genotype interaction we observed. We only used three different isolates of *H. defensa*, all obtained from *A. f. fabae*, and found that the rate of successful parasitism depended strongly on the combination of *H. defensa* isolate and parasitoid genotype. More specifically, parasitoids well adaptated to *H. defensa* isolate H323 were also well adapted to isolate H402, but not to isolate H76, and vice-versa. These results indicate that parasitoids may not be able to adapt to the general presence of *H. defensa* in their hosts, because they would have to adapt to the specific strains of *H. defensa* harboured by their local hosts. To address this, parasitoids would have to be tested on sympatric and allopatric isolates of *H. defensa* within the same host genetic backgrounds, which would be a difficult experiment to perform.

Another potential explanation for the lack of parasitoid local adapation is related to the relative migration rates of hosts and parasitoids. Studies on parasites with higher migration rates than their hosts report local adaptation, as measured by infection success, significantly more often than studies of parasites with relatively low migration rates [63], which is also supported by models [64]. Although the dispersal rate of *L. fabarum* is unknown, other species of this genus were found to be relatively poor dispersers [65, 66]. *Aphis f. fabae*, on the other hand, seems to exhibit rather high dispersal rates [67]. Hence, the evolution of parasitoid local adaptation may be hampered by *L. fabarum* exhibiting a low dispersal rate compared to its hosts.

A global pattern of local adaptation may also be obscured by time-lags of parasitoid adaptation [68, 69], or by the fact that our estimates of *H. defensa* prevalence per site did not accurately reflect the prevalence experienced by parasitoids. All five aphid species we considered grow on weedy plants that are very common across the study area, but they are of course unlikely to be equally common at each particular site. As a consequence, relative population sizes of the five aphid species must have varied among sites, and because species differed strongly in their infection with *H. defensa*, this would in turn have affected the local prevalence experienced by parasitoids. Since it was impossible to estimate the different aphids’ population sizes at each site, this additional variation was not captured by our estimate of *H. defensa*’s local prevalence, which was simply an unweighted average of the local prevalences in the five species.

Although parasitoids collected from different aphid host species exhibited significant differences in their ability to overcome *H. defensa*-mediated defences, there was no indication that parasitoids collected from host species with a high frequency of infection by *H. defensa* were better adapted to symbiont-conferred resistance. For example, despite the very low frequency of *H. defensa* in *A. f. cirsiiacanthoides*, parasitoids collected from this host exhibited the highest average infectivity on *H. defensa*-bearing aphids in our experiment (Fig. 2). This outcome should be interpreted with caution, however, because of the limited number of *H. defensa* isolates used for the tests and the strong parasitoid-by-*H. defensa* specificity reported above. Work on pea aphids indicates that host races associated with different plants tend to harbour different genotypes of *H. defensa* [57, 59], thus the same might apply to the different *Aphis* species investigated here. Unfortunately, we still know very little about the genetic structure of *H. defensa* across different host species in the field. If different lines of *L. fabarum* preferentially exploited particular host species, for which there is some evidence from the genetic structure of natural populations [47], and if parasitoids were adapted to strains of *H. defensa* specific to particular aphid hosts, this would have been impossible to detect with the design of the present experiment.

## Conclusions

To summarize, this study is the first, to our knowledge, to explicitly test for local adaptation of parasitoids to the frequency of protective symbionts in their hosts. While it yielded useful information on the occurrence of facultative endosymbionts in several important host species of *L. fabarum*, it provided only very limited evidence, at best, that this parasitoid is more infective on protected hosts when it comes from sites with a high prevalence of the protective symbiont *H. defensa*. It is worth mentioning here that an ‘inadvertent’ test of the same hypothesis was already published in 2001 by Hufbauer [70], in a study on *Aphidius ervi*, a parasitoid of pea aphids. Pea aphids specialized on alfalfa are much more resistant to this parasitoid than pea aphids specialized on clover [12], which could be related retrospectively to the fact that the alfalfa host race of the pea aphid is more frequently infected with *H. defensa* than the clover host race [e.g. 56]. Hufbauer [70] found that parasitoids from alfalfa fields were not more successful in parasitizing resistant pea aphids from alfalfa than parasitoids from clover fields, an outcome that is consistent with the negative result we report here. However, due to the limitations we discussed above, our negative result should not be taken as evidence that aphid parasitoids generally do not show local adaptation to their hosts’ endosymbionts. To better address this issue, the presumed variation of *H. defensa* genotypes among geographic locations and aphid species will have to be incorporated in the experiments, which will be very challenging, but not impossible. Finally, by using different isolates of *H. defensa* within a single aphid clone, we confirmed that the high specificity in the interaction between aphids harbouring *H. defensa* and their parasitoids, which was also reported in previous studies [23, 40, 62], is mediated by the symbiont’s genotype, rather than the host’s.

## Additional files

Additional file 1: Table S1. Collection information for aphid samples reported in the study with numbers of individuals per species and site screened successfully for the presence of five facultative endosymbionts. (DOCX 17 kb)
Additional file 2: Table S2. Collection information for parasitoid samples reported in the study with numbers of parasitoid lines per site and aphid host species that could be established successfully in the laboratory and tested for the ability to overcome *Hamiltonella defensa*-conferred resistance. (DOCX 17 kb)
Additional file 3: Table S3. List of parasitoid lines used in the experiment that showed identical microsatellite genotypes and were thus inferred to belong to the same asexual lineages of *Lysiphlebus fabarum*. (DOCX 16 kb)
Additional file 4: Table S4. Proportion of individuals infected with *Hamiltonella defensa* in samples of five aphid species collected from 17 sites in Switzerland and France. (DOCX 16 kb)
