# Supplementary material for: Are aphid parasitoids locally adapted to the prevalence of defensive symbionts in their hosts?
Source: BMC Evol Biol. 2016 Dec 12;16:271. doi: 10.1186/s12862-016-0811-0 (PMC5153875; doi:10.1186/s12862-016-0811-0)
Supplement: Additional file 1: Table S1. — Collection information for aphid samples reported in the study with numbers of individuals per species and site screened successfully for the presence of five facultative endosymbionts. (DOCX 17 kb) [file 12862_2016_811_MOESM1_ESM.docx]

**Additional File 1:**

**Table S1.** Collection information for aphid samples reported in the study with numbers of individuals per species and site screened successfully for the presence of five facultative endosymbionts.

| Site | Lat./Long. | Date | *A. f. cirsiiacanthoides* | *A. f. fabae* | *A. hederae* | *A. ruborum* | *A. urticata* |
| --- | --- | --- | --- | --- | --- | --- | --- |
| Aesch, CH | 47°28′N/7°35′E | 24/6/2009 | 17 | 28 | 22 | 20 | 22 |
| Alpnach, CH | 46°56′N/8°16′E | 9/7/2009 | 0* | 21 | 16 | 21 | 17 |
| Chur, CH | 46°51′N/9°32′E | 28/5/2009 | 23 | 20 | 18 | 17 | 22 |
| Geneva, CH | 46°12′N/6°09′E | 25/6/2009 | 20 | 30 | 24 | 13 | 22 |
| Grosses Moos, CH | 47°00′N/7°60′E | 18/6/2009 | 17 | 22 | 22 | 16 | 9 |
| Langenthal, CH | 47°13′N/7°47′E | 3/7/2009 | 20 | 22 | 23 | 9 | 20 |
| Magadino, CH | 46°09′N/8°51′E | 25/5/2009 | 0* | 20 | 16 | 1* | 20 |
| Martigny, CH | 46°06’N/7°40′E | 1/6/2009 | 25 | 25 | 18 | 5 | 16 |
| Mendrisio, CH | 45°52′N/8°59′E | 25/5/2009 | 1* | 27 | 11 | 29 | 20 |
| Montélimar, F | 44°33′N/4°45′E | 20-21/5/2009 | 34 | 24 | 16 | 13 | 13 |
| Neunkirch, CH | 47°41′N/8°29′E | 14/6/2009 | 22 | 32 | 22 | 22 | 24 |
| Orbe, CH | 46°43′N/6°32′E | 25/6/2009 | 18 | 27 | 14 | 15 | 22 |
| Remoulins, F | 43°56′N/4°33′E | 21/5/2009 | 17 | 28 | 10 | 13 | 13 |
| Romans, F | 45°02′N/5°03′E | 21-22/5/2009 | 21 | 29 | 11 | 19 | 23 |
| Sierre, CH | 46°18′N/7°32′E | 1/6/2009 | 16 | 24 | 22 | 10 | 20 |
| St. Margrethen, CH | 47°27′N/9°38′E | 1/7/2009 | 24 | 25 | 24 | 19 | 22 |
| Zurich, CH | 47°22′N/8°33′E | 12-13/6/2009 | 15 | 5 | 20 | 16 | 23 |

*Missing or very small samples (1 individual) were omitted before calculation of averages reported in the paper, and the affected sites (Alpnach, Mendrision and Magadino) were excluded from the analyses reported in Table 1.
