# Supplementary material for: Are aphid parasitoids locally adapted to the prevalence of defensive symbionts in their hosts?
Source: BMC Evol Biol. 2016 Dec 12;16:271. doi: 10.1186/s12862-016-0811-0 (PMC5153875; doi:10.1186/s12862-016-0811-0)
Supplement: Additional file 2: Table S2. — Collection information for parasitoid samples reported in the study with numbers of parasitoid lines per site and aphid host species that could be established successfully in the laboratory and tested for the ability to overcome Hamiltonella defensa-conferred resistance. (DOCX 17 kb) [file 12862_2016_811_MOESM2_ESM.docx]

**Additional File 2:**

**Table S2.** Collection information for parasitoid samples reported in the study with numbers of parasitoid lines per site and aphid host species that could be established successfully in the laboratory and tested for the ability to overcome *Hamiltonella defensa*-conferred resistance.

| Site | Lat./Long. | Date | *A. f. cirsiiacanthoides* | *A. f. fabae* | *A. hederae* | *A. ruborum* | *A. urticata* |
| --- | --- | --- | --- | --- | --- | --- | --- |
| Aesch, CH | 47°28′N/7°35′E | 24/6/2009 | 0 | 1 | 1 | 4 | 1 |
| Alpnach, CH | 46°56′N/8°16′E | 9/7/2009 | 0 | 0 | 2 | 3 | 2 |
| Chur, CH | 46°51′N/9°32′E | 28/5/2009 | 0 | 0 | 0 | 0 | 1 |
| Geneva, CH | 46°12′N/6°09′E | 25/6/2009 | 5 | 1 | 0 | 1 | 0 |
| Grosses Moos, CH | 47°00′N/7°60′E | 18/6/2009 | 2 | 0 | 3 | 0 | 0 |
| Langenthal, CH | 47°13′N/7°47′E | 3/7/2009 | 2 | 1 | 1 | 0 | 0 |
| Magadino, CH | 46°09′N/8°51′E | 25/5/2009 | 0 | 3 | 1 | 0 | 3 |
| Martigny, CH | 46°06’N/7°40′E | 1/6/2009 | 2 | 3 | 3 | 0 | 1 |
| Mendrisio, CH | 45°52′N/8°59′E | 25/5/2009 | 0 | 2 | 0 | 0 | 1 |
| Montélimar, F | 44°33′N/4°45′E | 20-21/5/2009 | 1 | 1 | 1 | 0 | 0 |
| Neunkirch, CH | 47°41′N/8°29′E | 14/6/2009 | 1 | 0 | 2 | 2 | 2 |
| Orbe, CH | 46°43′N/6°32′E | 25/6/2009 | 5 | 3 | 2 | 2 | 0 |
| Remoulins, F | 43°56′N/4°33′E | 21/5/2009 | 0 | 0 | 1 | 0 | 0 |
| Romans, F | 45°02′N/5°03′E | 21-22/5/2009 | 2 | 1 | 1 | 0 | 3 |
| Sierre, CH | 46°18′N/7°32′E | 1/6/2009 | 2 | 1 | 1 | 0 | 3 |
| St. Margrethen, CH | 47°27′N/9°38′E | 1/7/2009 | 3 | 0 | 1 | 0 | 2 |
| Zurich, CH | 47°22′N/8°33′E | 12-13/6/2009 | 1 | 0 | 6 | 0 | 3 |
