# Supplementary material for: Are aphid parasitoids locally adapted to the prevalence of defensive symbionts in their hosts?
Source: BMC Evol Biol. 2016 Dec 12;16:271. doi: 10.1186/s12862-016-0811-0 (PMC5153875; doi:10.1186/s12862-016-0811-0)
Supplement: Additional file 3: Table S3. — List of parasitoid lines used in the experiment that showed identical microsatellite genotypes and were thus inferred to belong to the same asexual lineages of Lysiphlebus fabarum. (DOCX 16 kb) [file 12862_2016_811_MOESM3_ESM.docx]

**Additional File 3:**

**Table S3.** List of parasitoid lines used in the experiment that showed identical microsatellite genotypes and were thus inferred to belong to the same asexual lines of *Lysiphlebus fabarum*.

| Genotype ID | Parasitoid line | Site | Host aphid |
| --- | --- | --- | --- |
| L01 | 09-120 | Mendrisio, CH | *A. urticata* |
|  | 09-171 | Magadino, CH | *A. f. fabae* |
| L02 | 09-155 | Magadino, CH | *A. urticata* |
|  | 09-541 | Aesch, CH | *A. urticata* |
|  | 09-565 | St. Margrethen, CH | *A. urticata* |
| L03 | 09-159 | Magadino, CH | *A. urticata* |
|  | 09-525 | Neunkirch, CH | *A. urticata* |
| L04 | 09-258 | Zurich, CH | *A. hederae* |
|  | 09-260 | Zurich, CH | *A. hederae* |
| L05 | 09-276 | Zurich, CH | *A. urticata* |
|  | 09-285 | Zurich, CH | *A. urticata* |
|  | 09-403 | Alpnach, CH | *A. ruborum* |
| L06 | 09-348 | Geneva, CH | *A. f. cirsiiacanthoides* |
|  | 09-381 | Orbe, CH | *A. f. cirsiiacanthoides* |
| L07 | 09-405 | Alpnach, CH | *A. urticata* |
|  | 09-407 | Alpnach, CH | *A. urticata* |
