# Supplementary material for: Are aphid parasitoids locally adapted to the prevalence of defensive symbionts in their hosts?
Source: BMC Evol Biol. 2016 Dec 12;16:271. doi: 10.1186/s12862-016-0811-0 (PMC5153875; doi:10.1186/s12862-016-0811-0)
Supplement: Additional file 4: Table S4. — Proportion of individuals infected with Hamiltonella defensa in samples of five aphid species collected from 17 sites in Switzerland and France. (DOCX 16 kb) [file 12862_2016_811_MOESM4_ESM.docx]

**Additional File 4:**

**Table S4.** Proportion of individuals infected with *H. defensa* in samples of five aphid species collected from 17 sites in Switzerland (CH) and France (F). Sample sizes are reported in Additional file 1: Table S1.

| Site | *A. f. cirsiiacanthoides* | *A. f. fabae* | *A. hederae* | *A. ruborum* | *A. urticata* | Site average across hosts |
| --- | --- | --- | --- | --- | --- | --- |
| Aesch, CH | 0.000 | 0.536 | 0.318 | 0.950 | 0.545 | 0.470 |
| Alpnach, CH | NA (0.048)* | 0.571 | 0.125 | 0.857 | 0.353 | 0.391 |
| Chur, CH | 0.000 | 0.450 | 0.389 | 0.882 | 0.091 | 0.362 |
| Geneva, CH | 0.050 | 0.567 | 0.000 | 0.692 | 0.500 | 0.362 |
| Grosses Moos, CH | 0.000 | 0.318 | 0.409 | 1.000 | 0.222 | 0.390 |
| Langenthal, CH | 0.050 | 0.682 | 0.174 | 0.667 | 0.400 | 0.394 |
| Magadino, CH | NA (0.048)* | 0.300 | 0.000 | NA (0.750)* | 0.000 | 0.220 |
| Martigny, CH | 0.040 | 0.240 | 0.222 | 0.800 | 0.250 | 0.310 |
| Mendrisio, CH | NA (0.048)* | 0.370 | 0.091 | 0.552 | 0.050 | 0.222 |
| Montélimar, F | 0.176 | 0.875 | 0.188 | 0.615 | 0.154 | 0.402 |
| Neunkirch, CH | 0.000 | 0.313 | 0.318 | 0.955 | 0.125 | 0.342 |
| Orbe, CH | 0.000 | 0.185 | 0.286 | 0.600 | 0.045 | 0.223 |
| Remoulins, F | 0.235 | 0.964 | 0.500 | 0.769 | 0.000 | 0.494 |
| Romans, F | 0.000 | 0.966 | 0.091 | 0.579 | 0.391 | 0.405 |
| Sierre, CH | 0.125 | 0.250 | 0.591 | 0.600 | 0.200 | 0.353 |
| St. Margrethen, CH | 0.000 | 0.520 | 0.250 | 0.737 | 0.227 | 0.347 |
| Zurich, CH | 0.000 | 0.600 | 0.450 | 0.750 | 0.130 | 0.386 |

*Missing estimates because samples were lacking or too small (only 1 individual). Missing estimates were replaced by species means (in brackets) for the calculation of site averages across hosts.
